# Attitudes and Perceptions Toward COVID-19 Digital Surveillance: Survey of Young Adults in the United States

**DOI:** 10.2196/23000

**Published:** 2021-01-08

**Authors:** Lauren Maytin, Jason Maytin, Priya Agarwal, Anna Krenitsky, JoAnn Krenitsky, Robert S Epstein

**Affiliations:** 1 Epstein Health LLC Woodcliff Lake, NJ United States

**Keywords:** attitude, perception, young adult, COVID-19, digital surveillance, population health technologies, surveillance, population, survey, adolescent

## Abstract

**Background:**

COVID-19 is an international health crisis of particular concern in the United States, which saw surges of infections with the lifting of lockdowns and relaxed social distancing. Young adults have proven to be a critical factor for COVID-19 transmission and are an important target of the efforts to contain the pandemic. Scalable digital public health technologies could be deployed to reduce COVID-19 transmission, but their use depends on the willingness of young adults to participate in surveillance.

**Objective:**

The aim of this study is to determine the attitudes of young adults regarding COVID-19 digital surveillance, including which aspects they would accept and which they would not, as well as to determine factors that may be associated with their willingness to participate in digital surveillance.

**Methods:**

We conducted an anonymous online survey of young adults aged 18-24 years throughout the United States in June 2020. The questionnaire contained predominantly closed-ended response options with one open-ended question. Descriptive statistics were applied to the data.

**Results:**

Of 513 young adult respondents, 383 (74.7%) agreed that COVID-19 represents a public health crisis. However, only 231 (45.1%) agreed to actively share their COVID-19 status or symptoms for monitoring and only 171 (33.4%) reported a willingness to allow access to their cell phone for passive location tracking or contact tracing.

**Conclusions:**

Despite largely agreeing that COVID-19 represents a serious public health risk, the majority of young adults sampled were reluctant to participate in digital monitoring to manage the pandemic. This was true for both commonly used methods of public health surveillance (such as contact tracing) and novel methods designed to facilitate a return to normal (such as frequent symptom checking through digital apps). This is a potential obstacle to ongoing containment measures (many of which rely on widespread surveillance) and may reflect a need for greater education on the benefits of public health digital surveillance for young adults.

## Introduction

The COVID-19 pandemic reached a disturbing milestone in the United States on November 21, 2020, as the number of confirmed cases surpassed 12 million, with the virus now spreading more rapidly and more broadly than ever before [1]. Since the summer of 2020, when states reopened businesses and public spaces, there has been a resurgence of cases as lockdowns were lifted and community spread intensified.

Young adults are believed to have played a major role in the increased number of cases and the heightened transmission of COVID-19 as social gatherings resumed and colleges and universities returned to campus [2]. Though hospitalization rates and mortality are currently lower for young adults than older adults [3], growing evidence suggests that younger generations are a major vector of COVID-19 transmission, comprising a relatively large proportion of the total confirmed cases [4]. Furthermore, COVID-19 incidence has increased among those aged 0-39 years as the pandemic has progressed, shifting the age distribution of cases over time from older to younger demographics [5].

While young adults play a significant role in the spread of COVID-19, they also tend to display a greater indifference toward the health risk posed by the pandemic and may be more resistant to policies aimed at reducing transmission. Young adults are significantly more likely to refuse a COVID-19 vaccine [6], less likely to closely follow COVID-19 news [7], and tend to see the pandemic as a greater risk to their finances than their health [8]. The relatively low mortality and severity of COVID-19 symptoms in this age group may contribute to this mindset. In addition, asymptomatic and mild cases comprise the majority of young COVID-19 cases and are believed to contribute significantly to community spread [9,10]. All of these factors (lower perceived vulnerability, reduced disease severity, higher engagement in social activities, and relatively high infection rate) made young adults a driving force of resurgences of COVID-19 [11].

As part of reopening plans, most states hired thousands of contact tracers to conduct public health surveillance and outreach to control and contain the spread of COVID-19 [12]. However, a recent study demonstrated that controlling the epidemic by manual contact tracing is not feasible given the infectiousness of COVID-19 and the high incidence of transmissions from presymptomatic or asymptomatic individuals [13]. These researchers proposed that an app providing instant digital contact tracing is needed for epidemic control. Beyond this kind of passive digital proximity and contact tracing, active symptom monitoring using mobile technology is also viewed as a key component for public health entities to better assess the community burden of COVID-19 [14]. Taken together, the active and passive surveillance of populations with digital public health tools has the potential to enable monitoring of COVID-19 status in real time and can be deployed rapidly and at scale, allowing targeted interventions to control spread.

To be successful, any COVID-19 precision public health control efforts that include digital surveillance must have a significant acceptance by the community. In particular, it is important to know how young adults would use these population health technologies for COVID-19 monitoring and whether they believe them to be necessary or beneficial. Therefore, we sought to understand the views of young adults regarding digital surveillance, including which aspects they would accept and which they would not, and define the factors that may influence their willingness to participate in digital monitoring of their movements or health status to help control the spread of COVID-19.

## Methods

### Study Design

The study was designed to be a national cross-sectional survey of young adults aged 18-24 years. Participants were recruited to answer an online questionnaire in which most questions were closed-ended by design, with 2-5 response options.

### Target Sample

To be representative of the target US young adult population (those aged 18-24 years), the sample size was determined to be 500, assuming 95% confidence intervals, a 5% margin of error, and a completion rate of approximately 80%. Age and gender balancing were prespecified to ensure these strata were generalizable to US census data. All US regions were targeted.

### Questionnaire Development

The survey instrument was designed to meet the specified study objectives. The survey scope and questionnaire inputs were based on a review of the limited COVID-19–related published literature available at that time [15,16]. In addition, 2 experts in the design and development of survey instruments for research reviewed the survey and provided additional comments and are noted in the acknowledgment section.

The online survey was designed so that a respondent was required to answer each question before they were directed to the next question. Respondents were not able to go back and change answers already entered. No identifying questions were asked and all survey responses were deidentified. Unanswered questions were not permitted, with the exception of the single open-ended question, which was optional.

Prior to the start of the survey, participants were provided introductory information that described COVID-19 and how it is transmitted. In addition, the concept of digital monitoring was defined and examples given. The language provided to the participants and the survey questions are included as a supplemental file (Multimedia Appendix 1).

### Study Population (Inclusion and Exclusion Criteria)

The inclusion criteria for the study population were participants aged 18-24 years, of all genders, and residing in any of the census regions of the United States. Participants who did not meet these inclusion criteria were excluded from completing the online survey.

### Survey Platform and Participant Recruitment 

This online survey was fielded and conducted using the SurveyMonkey platform [17]. SurveyMonkey panels are recruited from a database of over 2.5 million people in the United States. These panels are representative of a current, diverse online population that voluntarily joins the SurveyMonkey platform for survey research. All panelists share demographic information about themselves such as gender, age, and region, and other targeting attributes such as job type or technology usage.

SurveyMonkey balances its panels according to census data of age and gender. Panelist profiles are regularly refreshed to ensure respondent profiles are always current, and email and location verification is used to detect fraud and identify exclusions to prevent duplicate responses to the same survey. Ongoing panel calibration studies ensure response quality is on par with national benchmarks [18].

SurveyMonkey reaches panelists through technological means such as computer or mobile devices and offers a charitable incentive model. Panelists take surveys for charity and a chance to play a sweepstakes instant-win game. Panelists earn credits for completing surveys that they can redeem for gift cards or donate to charity [19].

### Data Management and Analysis

Deidentified survey responses were collected on the SurveyMonkey platform and exported for analysis. The data were aggregated to ensure anonymity and key findings were summarized using descriptive statistics. Survey respondents who disagreed with participating in any form of digital surveillance for COVID-19 were categorized and compared to all other respondents. Chi-square tests were used to calculate *P* values for categorical variables and *t* tests were used for comparing continuous variables.

### Ethical Considerations

Prior to patient recruitment and to comply with human subjects research requirements, we submitted our protocol and questionnaire to the Western Institutional Review Board. They determined the study was exempt under 45 CFR § 46.104(d)(2) because the research involved no more than minimal risk to subjects and only included interactions involving educational tests, survey procedures, interview procedures, or observations of public behavior.

Individuals aged 18-24 years were invited to participate and consented to participate via acceptance of a SurveyMonkey survey invitation. A brief introduction to the survey content was provided before participants opted to stop or continue to the question and answer portion of the survey. Each question of the survey included a “no response” option should the respondent prefer not to share that information. Respondents were allowed to withdraw from the survey at any time.

## Results

SurveyMonkey audience sampling identified 809 prescreened panelists who were invited to participate. A total of 548 respondents initiated the survey. Of these, 35 participants abandoned the survey and 513 completed it, for a survey completion rate of 93.6%. Nearly all (99.8%) respondents used a mobile phone or tablet to complete the questionnaire. Table 1 summarizes the sociodemographics of participants. With a mean age of 20.6 years, nearly two-thirds of participants had partially completed or completed college. When asked whether they knew someone who had contracted COVID-19 or if they had contracted it themselves, 192 (37.4%) answered in the affirmative.

**Table 1:** Sociodemographic characteristics of survey participants (N=513).

Variables		Values
Mean age, years (SD)		20.6 (2)
Age range, years		18-24
**Gender, n (%)**		
	Male	261 (51)
	Female	246 (48)
	Other	6 (1)
**Highest educational level, n (%)**		
	Some high school	27 (5.3)
	High school	134 (26)
	Some college	198 (38.6)
	College	121 (23.6)
	Graduate/professional degree	33 (6.4)
**Race/ethnicity, n (%)**		
	American Indian/Alaskan	6 (1.2)
	Asian	79 (15.4)
	Black/African American	60 (11.7)
	Hispanic/Latino	96 (18.7)
	Native Hawaiian	5 (1.0)
	White	228 (44.4)
	Multiethnic	39 (7)
**Geographic region of residence, n (%)**		
	Northeast	124 (24.2)
	Midwest	100 (19.5)
	Southeast	100 (19.5)
	South	80 (15.6)
	West	39 (7.6)
	Pacific	63 (12.3)
	Alaska or Hawaii	7 (1.4)
**Participant, close friend, and/or family had COVID-19, n (%)**		
	Yes	192 (37.4)
	No	321 (62.6)

Most (n=383, 74.7%) young adults agreed that the COVID-19 pandemic is a public health crisis that poses significant risk to the health and safety of the US population (Table 2). However, only 56.9% (n=292) agreed that digital monitoring would be effective in helping to stop COVID-19 transmission. Even fewer young adults (n=236; 46.0%) agreed that digital monitoring would be necessary for a return to normal. Over half of young adult participants expressed privacy concerns about personal information being used in digital surveillance systems for COVID-19 monitoring.

**Table 2:** Responses of young adults in the United States (N=513) to questions about their beliefs and concerns regarding COVID-19 and digital surveillance.

Beliefs/concerns and responses		Participants, n (%)
**Believes the COVID-19 pandemic is a public health crisis**		
	Strongly disagree	30 (5.8)
	Disagree	44 (8.6)
	Neutral	56 (10.9)
	Agree	186 (36.3)
	Strongly agree	197 (38.4)
**Believes digital tracking/monitoring would help stop the spread of COVID-19**		
	Strongly disagree	43 (8.9)
	Disagree	53 (10.3)
	Neutral	125 (24.4)
	Agree	188 (36.7)
	Strongly agree	104 (20.3)
**Believes digital tracking/monitoring is necessary to return to normal**		
	Strongly disagree	58 (11.3)
	Disagree	76 (14.8
	Neutral	143 (27.9)
	Agree	145 (28.3)
	Strongly agree	91 (17.8)
**Concerns about the privacy of my information used for tracking/monitoring**		
	Strongly disagree	42 (8.2)
	Disagree	85 (16.6)
	Neutral	126 (24.6)
	Agree	139 (27.1)
	Strongly agree	121 (23.6)

Young adults expressed differences in opinion regarding their willingness to participate in the two types of potential COVID-19 tracking. Nearly half (n=231; 45.1%) stated their willingness for active monitoring (eg, they would manually input data via their phone/tablet), while only 33.3% (n=171) stated a willingness for passive monitoring (eg, monitoring of location and contacts by cell phone; Table 3). Approximately 25% of responses to each question were neutral. Young adults also appeared more willing to share personal health information than location or contact data.

**Table 3:** Responses of young adults in the United States (N=513) to questions about their willingness to participate in aspects of COVID-19 digital surveillance.

Questions and responses		Participants, n (%)
**Willing to allow cell phone to passively monitor**		
	Strongly disagree	99 (19.3)
	Disagree	113 (22)
	Neutral	130 (25.3)
	Agree	121 (23.6)
	Strongly agree	50 (9.8)
**Willing to actively input specific health data via phone/tablet**		
	Strongly disagree	58 (11.9)
	Disagree	96 (18.7)
	Neutral	25 (24.4)
	Agree	167 (32.6)
	Strongly agree	64 (12.5)
**Willing to share results of any COVID-19 virus or antibody tests**		
	Strongly disagree	60 (11.7)
	Disagree	52 (10.1)
	Neutral	112 (21.8)
	Agree	200 (39)
	Strongly agree	89 (17.4)
**Willing to share symptom information such as coughing, tiredness, or temperature**		
	Strongly disagree	68 (13.3)
	Disagree	66 (12.9)
	Neutral	106 (20.7)
	Agree	193 (37.6)
	Strongly agree	80 (15.6)
**Willing to share my location and where I have been, tracked by my phone**		
	Strongly disagree	146 (28.5)
	Disagree	105 (20.5)
	Neutral	120 (23.4)
	Agree	103 (20.1)
	Strongly agree	39 (7.6)
**Willing to share personal contact data such as who I was with, tracked by my phone**		
	Strongly disagree	138 (26.9)
	Disagree	117 (22.8)
	Neutral	114 (22.2)
	Agree	99 (19.3)
	Strongly agree	45 (8.8)

When asked to select the entities with whom they would be willing to share their personal information (Table 4), 62.6% (n=321) of young adults endorsed sharing information with their doctor/health care provider. Government agencies (local and federal) were the next most trusted option, with a similar number willing to share their data with local or federal government agencies and researchers. Only about one-third of the respondents were willing to share their information with schools or employers. By far, the group that respondents trusted least with their information was private companies.

Respondents were asked whether they would agree to participate in monitoring prior to engaging in select activities to help control viral transmission (Table 5). None of the activities generated agreement from more than half of participants, and nearly one-fourth of respondents stated that they would not agree to any monitoring to participate in the listed activities.

There were no significant differences in mean age, gender, or education between those willing or neutral regarding being tracked for COVID-19 risk behaviors/symptoms versus those who were unwilling (Table 6). Significant differences were noted in race/ethnicity in univariate analyses.

**Figure 1:**
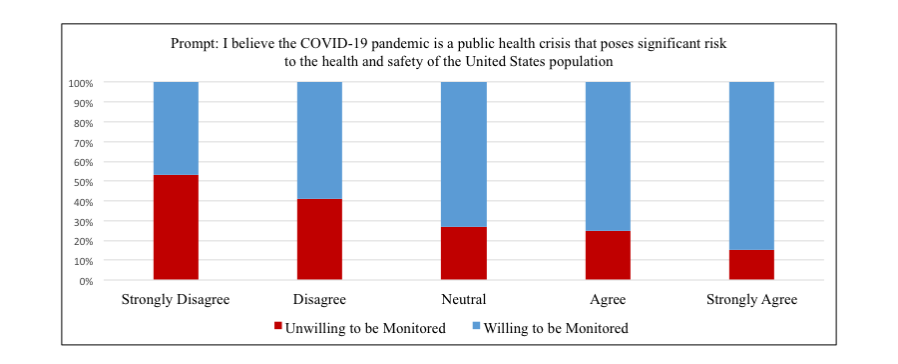
Relationship between belief in COVID-19 as a significant public health crisis and willingness to be digitally monitored from survey of young adults in the United States (N=513).

There was a stepwise relationship between disbelief that COVID-19 is a public health crisis and lack of willingness to participate in digital tracking (Figure 1). At the extremes, only 15.2% (30/197) of respondents who strongly agreed that COVID-19 is a public health crisis were unwilling to be tracked, whereas 53.3% (16/30) of respondents who strongly disagreed that COVID-19 is a public health crisis were unwilling to be tracked.

**Table 4:** Responses of young adults in the United States (N=513) to a question regarding their willingness to share personal health data with individuals or organizations for COVID-19 digital surveillance.

Potential data recipient	Willing to share information, n (%)
Your doctor or other health care provider responsible for your care	321 (62.6)
Local, county, or state health department	199 (38.8)
Federal agencies or researchers (such as the Centers for Disease Control and Prevention or the National Institutes of Health)	189 (36.8)
Your school	174 (33.9)
Your employer	156 (30.4)
Companies such as Google, Microsoft, Apple, and Facebook	78 (15.2)
None of these	76 (14.8)

**Table 5:** Responses of young adults in the United States (N=513) to a question regarding activities they would be willing to agree to COVID-19 digital surveillance prior to participating.

Activity	Willing participants, n (%)
Travel by airplane	222 (43.3)
Visit elderly or sick family members in a nursing home or hospital	189 (36.8)
Return to in-person attendance at school or work	183 (35.7)
Attend large social gatherings (eg, wedding, graduation, birthday party)	182 (35.5)
Shop at an indoor mall	176 (34.3)
Travel by public transportation (eg, bus, train, subway)	173 (33.7)
Dine indoors at a restaurant	166 (32.4)
Be a spectator at a sporting event, concert, or movie theatre	164 (32.0)
Be in public without a mask or face covering	158 (30.8)
Participate in organized team sporting events	150 (29.2)
Return to indoor places of worship	149 (29.0)
Gather with family and friends that do not live with you	142 (27.7)
None of these	118 (23.0)

**Table 6:** Characteristics of young adults in the United States (N=513) unwilling to be passively or actively digitally monitored for COVID-19.

Factors		All others (n=388)	Unwilling (n=125)	*P* value
Mean age, years (SD)		20.6 (4)	20.6 (4)	.69
Gender (female), n (%)		182 (47)	64 (51)	.59
**Location, n (%)**				.09
	Alaska/Hawaii	4 (1)	3 (2.4)	N/A^a^
	Midwest	77 (19.8)	23 (18.4)	N/A
	Northeast	96 (24.7)	28 (22.4)	N/A
	Pacific	48 (12.4)	15 (12)	N/A
	South	65 (16.7)	15 (12)	N/A
	Southeast	76 (19.6)	24 (19.2)	N/A
	West	22 (5.6)	17 (13.6)	N/A
**Highest education, n (%)**				.45
	Some high school	17 (4.3)	10 (0.1)	N/A
	High school	100 (25.8)	34 (27.2)	N/A
	Some college	149 (38.4)	49 (39.2)	N/A
	College	95 (24.4)	26 (20.8)	N/A
	Graduate school	27 (6.9)	6 (4.8)	N/A
**Race/ethnicity, n (%)**				.002
	American Indian/Alaska Native	3 (0.1)	3 (2.4)	N/A
	Asian	68 (17.5)	11 (8.8)	N/A
	Black/African American	53 (13.7)	7 (5.6)	N/A
	Hispanic/Latino or Spanish	72 (18.6)	24 (19.2)	N/A
	Multiethnic	23 (5.9)	16 (12.8)	N/A
	Native Hawaiian/Islander	5 (1.2)	5 (4)	N/A
	White	164 (42.3)	64 (51.2)	N/A
Close friend or family member with COVID-19, n (%)		140 (36.1)	52 (41.6)	.32

^a^N/A: not applicable.

## Discussion

Although population health technologies such as digital contact tracing and symptom surveillance have the potential to be instrumental in controlling the spread of COVID-19, the young adults in our survey were not in agreement about the necessity and effectiveness of these public health practices. Although the vast majority agreed that COVID-19 is a public health threat, they were nearly evenly divided in their opinions about digital monitoring being a viable solution.

This is in stark contrast to public health recommendations to rely on surveillance systems to safely participate in daily activities such as work or school during the pandemic. A report released by the Duke-Margolis Center for Health Policy recommends that a national surveillance system be established to control the ongoing COVID-19 pandemic and suppress future resurgences [20]. The authors assert that widespread serologic and diagnostic testing capabilities, routine data sharing, syndromic surveillance, and the use of digital tools and resources (particularly digital apps already in use by some health systems) will be essential for rapidly tracing and quarantining new cases and for ongoing COVID-19 surveillance.

However, our research indicates that a somewhat large and consistent percentage (20%-25%) of young adults feel neutral regarding questions of surveillance effectiveness or necessity and expressed privacy concerns related to digital surveillance. Young adults may be especially resistant to or ambivalent about digital monitoring efforts due to the increased importance of peer-group social interactions during this stage of development as well as underestimation of the risk involved in such behavior. Adolescent self-esteem and sense of identity is greatly influenced by socialization with peers, in ways that may make young adults reluctant to abide by strict distancing and monitoring guidelines [21]. There may be a need for education directed at this demographic on the usefulness of these precision public health tools for infection control.

We also sought to understand if there were differences between those who might endorse active digital surveillance (eg, inputting daily symptoms in a digital app) versus passive digital surveillance (eg, location tracking using mobile phone information). Respondents showed a clear preference for active monitoring, with almost 12% more respondents agreeing to active monitoring than passive monitoring. This was surprising given the popularity among young adults of various social media apps (such as Snapchat) that use passive location-tracking services [22]. It may be that active monitoring provides young adults with a sense of control over choosing the information they share. Passive monitoring (such as location tracking) may feel less transparent and more invasive.

This finding is similar to other research that has shown that many Americans believe that passive monitoring through cell phones would be ineffective against COVID-19 and such surveillance is unacceptable [23]. Distrust toward the government and concerns about security and privacy are the main barriers to adoption of digital surveillance tools to control COVID-19 [24]. Young Americans in particular have been shown to have lower levels of trust compared to older Americans. This lower interpersonal level of trust extends to institutions such as elected officials, police officers, the military, and other civic leaders [25], and perhaps provides context for why young adults may perceive contact-tracing efforts as an invasion of their privacy.

Our survey results underscore the trust and privacy concerns young adults have toward entities conducting digital surveillance and about the potential misuse of their personal data. Respondents frequently cited potential abuse of information as a major concern created by COVID-19 digital monitoring, including such open-ended remarks as “I do not trust anyone in power with this information” and “I worry about who would have access to the data and the potential for federal overreach.” Others reported privacy concerns with statements such as “Strong invasion of privacy,” “My information would be used for something else and sold to companies,” and similar sentiments.

An unexpected finding of our research was that respondents felt more comfortable sharing medical data such as symptoms and COVID-19 test results rather than information such as location or personal contacts. Moreover, they were most comfortable sharing this information with their physician or other health care provider responsible for their care and not public health authorities, schools, or other entities.

Most importantly, young adult respondents showed overwhelming distrust toward sharing these data with private companies listed in the questionnaire as Google, Apple, Microsoft, or Facebook, with only 15.2% agreeing to share their information with such entities. Pandemic surveillance programs developed by these private companies may be severely hindered by this distrust, regardless of their functionality. This is similar to research that suggests that private technology entities are the least likely source for which individuals would be willing to use surveillance apps and that there is no single, authoritative provider to which everyone would be willing to share the data necessary for digital tracking apps to be effective [26].

It may be that these trust, privacy, and personal data concerns will pose a significant challenge in convincing individuals to use digital tracking applications [27]. However, for young adults frustrated with social distancing policies, digital monitoring may provide something of a middle ground, allowing them to continue social activities while also granting some control over COVID-19 spread. However, our research indicated that even for social activities that may involve the risk of possible infection, young adults were not supportive of surveillance measures that could keep them or others safe. There was a lack of consensus regarding the types of activities for which it would be acceptable for young adults to be monitored prior to participating. Agreement to participate in digital monitoring was fairly low for all activities, even those such as visiting elderly family members in a hospital or nursing home, where the risk to themselves or others would presumably be greatest. There was no majority agreement on any one activity that would benefit from surveillance and 23% (n=118) of respondents were not willing to participate in surveillance for any activity.

This may support the idea that some individuals are unaware of the risks of their behaviors or underestimate their personal risk of infection relative to others [28]. Young adults have been shown to exhibit a higher willingness to accept risk in situations where the likelihood of positive or negative outcomes is unknown [29]. As demonstrated during the COVID-19 pandemic, this tendency manifested itself in continued socialization with peers despite social distancing guidelines, and a willingness to neglect public health safety measures such as mask wearing and handwashing when compared to older adults [30].

It is for this very reason (the underestimation of health risk) that the use of digital public health tools for COVID-19 is needed. The utility of population health technologies for disease surveillance has been shown in the past in studies of the transmission of influenza-like illness [31]. Radin et al [31] found that wearable device data significantly improved predictions of influenza-like illness transmission and concluded that such data collection systems could be crucial in guiding responses to suppress future outbreaks. For young adults, digital monitoring could be essential for a safe “return to normalcy,” but digital public health technologies’ effectiveness against COVID-19 will be dependent on widespread trust and uptake [32].

In addition, agencies cannot implement surveillance programs without a better understanding of obstacles to their success. Although many digital health tools have been rapidly deployed in response to the COVID-19 crisis, continuous improvements, modifications, and customizations will have to be made to these tactics so they may be personalized for the various populations they are meant to protect. Such tactics must take into account that young adults have been more likely to experience job or wage loss because of the COVID-19 outbreak and are more likely to report high levels of emotional distress during the COVID-19 pandemic compared to older Americans [8]. These impacts may be motivating the behaviors of young adults during this pandemic and influencing their attitudes toward surveillance efforts. Further research is needed on how young adults perceive their behavior and risks of COVID-19 to inform the future use of digital health technologies to monitor and control this disease in this population. For young adults, it may be that more education about the benefits of such precision public health efforts and the involvement of their trusted health care providers would be a path worth exploring to achieve digital surveillance goals.

One limitation of this study is that the sample population is not a random sample of the United States. The survey was conducted using the SurveyMonkey platform, where all of the respondents previously agreed to survey participation and should thus be viewed as a convenience sample. However, the age, gender, and ethnic and racial distribution of our survey participants is representative of the general young adult population in the United States [33]. The regional distribution of responses is also relatively representative of the US population, with the highest proportion residing in the Northeast (n=124; 24.2%). An additional limitation is that due to the rapidly changing nature of the pandemic, new information becoming available, and case numbers and personal circumstances changing, the findings of this research may not be reflective of shifting opinions.

In conclusion, despite largely agreeing that COVID-19 represents a serious public health risk, a large proportion of young adults are reluctant to participate in digital monitoring to manage the pandemic. This is true for both commonly used methods of public health surveillance (such as contact tracing) and novel methods designed to facilitate a return to normal (such as frequent symptom checking through digital apps). As a major vector of COVID-19 transmission, the participation of young adults in digital COVID-19 monitoring is important to its success. Ultimately, the hesitancy of young adults to participate in digital monitoring must be addressed for these public health surveillance systems to be effective.

## Appendix

Multimedia Appendix 1 Questionnaire used to survey young adults in the United States regarding COVID-19 digital surveillance.
