# Supplementary material for: Attitudes and Perceptions Toward COVID-19 Digital Surveillance: Survey of Young Adults in the United States
Source: JMIR Form Res. 2021 Jan 8;5(1):e23000. doi: 10.2196/23000 (PMC7800905; doi:10.2196/23000)
Supplement: Multimedia Appendix 1 [file formative_v5i1e23000_app1.pdf]

Survey of Young Adult Perceptions and Attitudes of COVID-19 Digital Surveillance

Overview
COVID-19 is a respiratory disease caused by a viral infection. It can be transmitted through speaking, coughing or sneezing when in close contact with people who have the disease but may or may not have symptoms. "Close contact" involves direct contact with a person's respiratory secretions.

Currently, there is no vaccine available to help stop the spread of COVID-19. Therefore, many areas in the United States mandated "stay-at-home" orders to limit close contact of individuals and help decrease infections.

Some experts believe lifting stay-at-home orders to return to a more normal way of life will involve digital monitoring. This means using technology to gather information about people. For example, location tracking through a cell phone or health monitoring with an app. It is believed that digital monitoring will keep infection rates low and prevent future outbreaks.

The following survey is designed to understand your beliefs and opinions regarding COVID-19 digital monitoring and tracking.

Instructions: Please select the option that best answers the question. These questions will help us better understand who is taking this survey.

1. What is your age?

- 18 years old
- 19 years old
- 20 years old
- 21 years old
- 22 years old
- 23 years old
- 24 years old

2. What is your gender?

- Female
- Male
- Other

3. What is the highest education level you have completed?

- Some high school
- High school
- Some college
- College Graduate/Professional

4. How would you best describe yourself?

- American Indian or Alaska Native
- Asian
- Black or African American
- Hispanic, Latino, or of Spanish origin
- Native Hawaiian or Other Pacific Islander
- White
- Multi-ethnic (two or more of the above)

5. In what area of the United States do you live?

- Northeast (Maine, New Hampshire, Vermont, Massachusetts, Rhode Island, Connecticut, New York, Pennsylvania, New Jersey)
- Midwest (Wisconsin, Michigan, Illinois, Indiana, Ohio, North Dakota, South Dakota, Nebraska, Kansas, Minnesota, Iowa, Missouri)
- Southeast (Delaware, Maryland, District of Columbia, Virginia, West Virginia, North Carolina, South Carolina, Georgia, Florida) South (Kentucky, Tennessee, Mississippi, Alabama, Oklahoma, Texas, Arkansas, Louisiana)
- West (Idaho, Montana, Wyoming, Nevada, Utah, Colorado, Arizona, New Mexico)
- Pacific (Washington, Oregon, California)
- Alaska or Hawaii

6. Have you or a close friend or family member ever been diagnosed with COVID-19?

- Yes
- No

Instructions: Please respond to each statement as to how much you disagree or agree.

7. I believe the COVID-19 pandemic is a public health crisis that poses significant risk to the health and safety of the United States population.

- Strongly Disagree
- Disagree
- Neither Disagree or Agree
- Agree
- Strongly Agree

8. I believe COVID-19 digital monitoring and tracking would be effective in helping to stop the spread of COVID-19 in the United States.

- Strongly Disagree
- Disagree
- Neither Disagree or Agree
- Agree
- Strongly Agree

9. I believe COVID-19 digital monitoring and tracking of the population is necessary in order for the United States to return to a more normal way of life.

- Strongly Disagree
- Disagree
- Neither Disagree or Agree
- Agree
- Strongly Agree

10. I have concerns about the privacy of my personal information being used in COVID-19 digital monitoring.

- Strongly Disagree
- Disagree
- Neither Disagree or Agree
- Agree
- Strongly Agree

11. I would be willing to participate in monitoring that can be accomplished through tracking my cell phone and requires little input on my part.

- Strongly Disagree
- Disagree
- Neither Disagree or Agree
- Agree
- Strongly Agree

12. I would be willing to participate in monitoring that requires me to report regular updates about my health by using a smart phone or tablet app.

- Strongly Disagree
- Disagree
- Neither Disagree or Agree
- Agree
- Strongly Agree

13. I would be willing to share or have others monitor my personal information related to:

a. Results of any COVID- 19 disease or antibody tests

- Strongly Disagree
- Disagree
- Neither Disagree or Agree
- Agree
- Strongly Agree

b. Any symptoms such as coughing, tiredness or temperature

- Strongly Disagree
- Disagree
- Neither Disagree or Agree
- Agree
- Strongly Agree

c. My location and where I have been, tracked by my phone

- Strongly Disagree
- Disagree
- Neither Disagree or Agree
- Agree
- Strongly Agree

d. Personal contact data such as who I was with, tracked by my phone

- Strongly Disagree
- Disagree
- Neither Disagree or Agree
- Agree
- Strongly Agree

Instructions: Please select all that apply.

14. With whom are you comfortable sharing your personal data to help them monitor and manage the spread of COVID-19? (Select all that apply.)

- Your doctor or other healthcare provider responsible for your care
- Your school
- Your employer
- Local, county or state health department
- Federal agencies or researchers (such as the Centers for Disease Control (CDC) or the National Institutes of Health (NIH))
- Companies such as Google, Microsoft, Apple, Facebook
- None of these

15. Where do you agree with the use of personal data to monitor and manage the spread of COVID-19? (Select all that apply.)

- Anonymous tracking of the number of cases of COVID-19 infections
- Alerting someone if they have unknowingly come into contact with a person diagnosed with COVID-19
- Providing personalized healthcare advice to someone who is sick with COVID-19
- Alerting officials when a person with COVID-19 does not follow stay-at-home orders
- None of these

16. For which of these activities would you agree to COVID-19 digital monitoring prior to participating? (Select all that apply.)

- Return to in-person attendance at school or work
- Return to indoor places of worship
- Gather with family and friends that do not live with you
- Visit elderly or sick family members in a nursing home or hospital
- Travel by public transportation such as a bus, train, or subway
- Travel by airplane
- Be in public without a mask or face covering
- Attend large social gatherings such as a wedding, graduation, or birthday party
- Participate in organized team sporting events
- Be a spectator at a sporting event, concert, or movie theatre
- Dine indoors at a restaurant
- Go shopping at an indoor mall
- None of these

17. Briefly share any additional comments or concerns you have about participating in COVID-19 digital monitoring.
